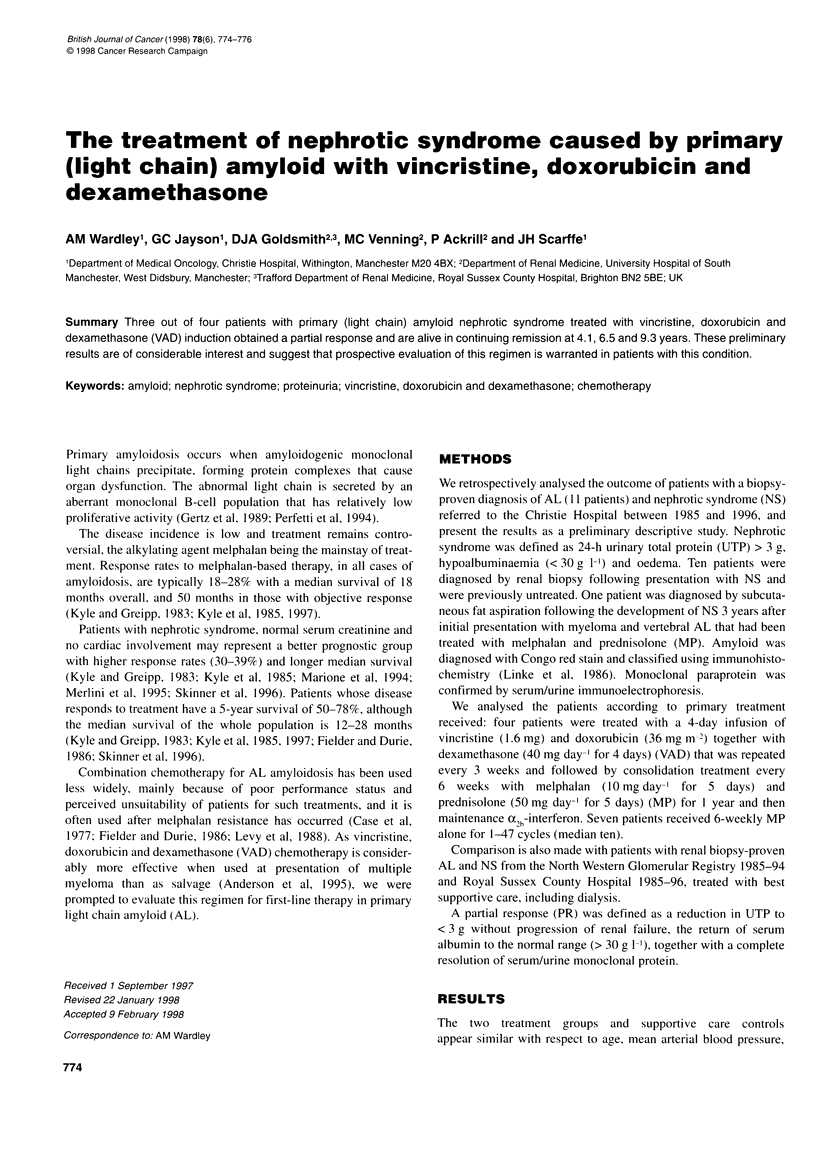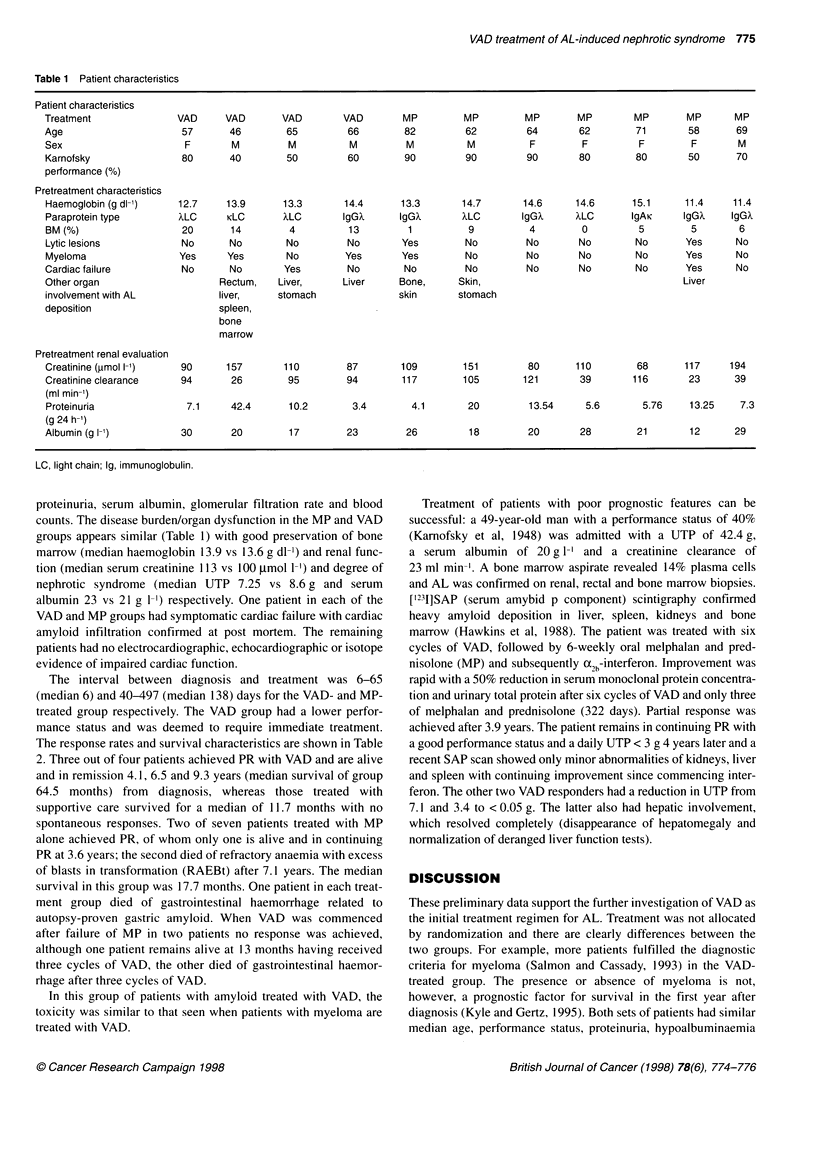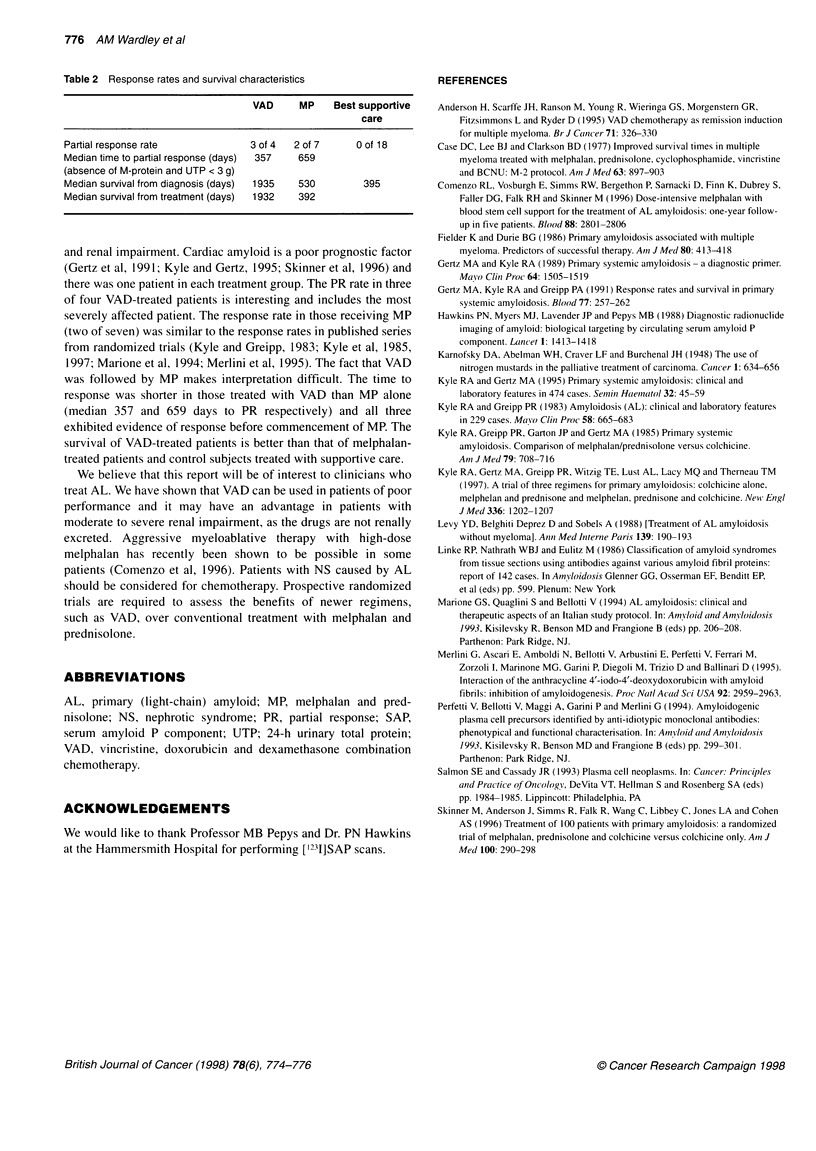# The treatment of nephrotic syndrome caused by primary (light chain) amyloid with vincristine, doxorubicin and dexamethasone.

**DOI:** 10.1038/bjc.1998.577

**Published:** 1998-09

**Authors:** A. M. Wardley, G. C. Jayson, D. J. Goldsmith, M. C. Venning, P. Ackrill, J. H. Scarffe

**Affiliations:** Department of Medical Oncology, Christie Hospital, Withington, Manchester, UK.

## Abstract

Three out of four patients with primary (light chain) amyloid nephrotic syndrome treated with vincristine, doxorubicin and dexamethasone (VAD) induction obtained a partial response and are alive in continuing remission at 4.1, 6.5 and 9.3 years. These preliminary results are of considerable interest and suggest that prospective evaluation of this regimen is warranted in patients with this condition.


British Journal of Cancer (1998) 78(6), 774–776
© 1998 Cancer Research Campaign

### The treatment of nephrotic syndrome caused by primary (light chain) amyloid with vincristine, doxorubicin and dexamethasone

**AM Wardley[1], GC Jayson[1], DJA Goldsmith[2,3], MC Venning[2], P Ackrill[2] and JH Scarffe[1]**

[1]Department of Medical Oncology, Christie Hospital, Withington, Manchester M20 4BX; [2]Department of Renal Medicine, University Hospital of South Manchester, West Didsbury, Manchester; [3]Trafford Department of Renal Medicine, Royal Sussex County Hospital, Brighton BN2 5BE; UK

**Summary** Three out of four patients with primary (light chain) amyloid nephrotic syndrome treated with vincristine, doxorubicin and dexamethasone (VAD) induction obtained a partial response and are alive in continuing remission at 4.1, 6.5 and 9.3 years. These preliminary results are of considerable interest and suggest that prospective evaluation of this regimen is warranted in patients with this condition.

**Keywords:** amyloid; nephrotic syndrome; proteinuria; vincristine, doxorubicin and dexamethasone; chemotherapy

Primary amyloidosis occurs when amyloidogenic monoclonal light chains precipitate, forming protein complexes that cause organ dysfunction. The abnormal light chain is secreted by an aberrant monoclonal B-cell population that has relatively low proliferative activity (Gertz et al, 1989; Perfetti et al, 1994).

The disease incidence is low and treatment remains controversial, the alkylating agent melphalan being the mainstay of treatment. Response rates to melphalan-based therapy, in all cases of amyloidosis, are typically 18–28% with a median survival of 18 months overall, and 50 months in those with objective response (Kyle and Greipp, 1983; Kyle et al, 1985, 1997).

Patients with nephrotic syndrome, normal serum creatinine and no cardiac involvement may represent a better prognostic group with higher response rates (30–39%) and longer median survival (Kyle and Greipp, 1983; Kyle et al, 1985; Marione et al, 1994; Merlini et al, 1995; Skinner et al, 1996). Patients whose disease responds to treatment have a 5-year survival of 50–78%, although the median survival of the whole population is 12–28 months (Kyle and Greipp, 1983; Kyle et al, 1985, 1997; Fielder and Durie, 1986; Skinner et al, 1996).

Combination chemotherapy for AL amyloidosis has been used less widely, mainly because of poor performance status and perceived unsuitability of patients for such treatments, and it is often used after melphalan resistance has occurred (Case et al, 1977; Fielder and Durie, 1986; Levy et al, 1988). As vincristine, doxorubicin and dexamethasone (VAD) chemotherapy is considerably more effective when used at presentation of multiple myeloma than as salvage (Anderson et al, 1995), we were prompted to evaluate this regimen for first-line therapy in primary light chain amyloid (AL).

*Received 1 September 1997*
*Revised 22 January 1998*
*Accepted 9 February 1998*

*Correspondence to:* AM Wardley

### METHODS

We retrospectively analysed the outcome of patients with a biopsy-proven diagnosis of AL (11 patients) and nephrotic syndrome (NS) referred to the Christie Hospital between 1985 and 1996, and present the results as a preliminary descriptive study. Nephrotic syndrome was defined as 24-h urinary total protein (UTP) > 3 g, hypoalbuminaemia (< 30 g $l^{-1}$) and oedema. Ten patients were diagnosed by renal biopsy following presentation with NS and were previously untreated. One patient was diagnosed by subcutaneous fat aspiration following the development of NS 3 years after initial presentation with myeloma and vertebral AL that had been treated with melphalan and prednisolone (MP). Amyloid was diagnosed with Congo red stain and classified using immunohistochemistry (Linke et al, 1986). Monoclonal paraprotein was confirmed by serum/urine immunoelectrophoresis.

We analysed the patients according to primary treatment received: four patients were treated with a 4-day infusion of vincristine (1.6 mg) and doxorubicin (36 mg $m^{-2}$) together with dexamethasone (40 mg $day^{-1}$ for 4 days) (VAD) that was repeated every 3 weeks and followed by consolidation treatment every 6 weeks with melphalan (10 mg $day^{-1}$ for 5 days) and prednisolone (50 mg $day^{-1}$ for 5 days) (MP) for 1 year and then maintenance $\alpha_{2b}$-interferon. Seven patients received 6-weekly MP alone for 1–47 cycles (median ten).

Comparison is also made with patients with renal biopsy-proven AL and NS from the North Western Glomerular Registry 1985–94 and Royal Sussex County Hospital 1985–96, treated with best supportive care, including dialysis.

A partial response (PR) was defined as a reduction in UTP to < 3 g without progression of renal failure, the return of serum albumin to the normal range (> 30 g $l^{-1}$), together with a complete resolution of serum/urine monoclonal protein.

### RESULTS

The two treatment groups and supportive care controls appear similar with respect to age, mean arterial blood pressure,

**Table 1** Patient characteristics

| | | | | | | | | | | | |
|---|---|---|---|---|---|---|---|---|---|---|---|
| Patient characteristics | | | | | | | | | | | |
| Treatment | VAD | VAD | VAD | VAD | MP | MP | MP | MP | MP | MP | MP |
| Age | 57 | 46 | 65 | 66 | 82 | 62 | 64 | 62 | 71 | 58 | 69 |
| Sex | F | M | M | M | M | M | F | F | F | F | M |
| Karnofsky performance (%) | 80 | 40 | 50 | 60 | 90 | 90 | 90 | 80 | 80 | 50 | 70 |
| Pretreatment characteristics | | | | | | | | | | | |
| Haemoglobin (g dl$^{-1}$) | 12.7 | 13.9 | 13.3 | 14.4 | 13.3 | 14.7 | 14.6 | 14.6 | 15.1 | 11.4 | 11.4 |
| Paraprotein type | λLC | κLC | λLC | IgGλ | IgGλ | λLC | IgGλ | λLC | IgAκ | IgGλ | IgGλ |
| BM (%) | 20 | 14 | 4 | 13 | 1 | 9 | 4 | 0 | 5 | 5 | 6 |
| Lytic lesions | No | No | No | No | Yes | No | No | No | No | Yes | No |
| Myeloma | Yes | Yes | No | Yes | Yes | No | No | No | No | Yes | No |
| Cardiac failure | No | No | Yes | No | No | No | No | No | No | Yes | No |
| Other organ involvement with AL deposition | | Rectum, liver, spleen, bone marrow | Liver, stomach | Liver | Bone, skin | Skin, stomach | | | | Liver | |
| Pretreatment renal evaluation | | | | | | | | | | | |
| Creatinine (μmol l$^{-1}$) | 90 | 157 | 110 | 87 | 109 | 151 | 80 | 110 | 68 | 117 | 194 |
| Creatinine clearance (ml min$^{-1}$) | 94 | 26 | 95 | 94 | 117 | 105 | 121 | 39 | 116 | 23 | 39 |
| Proteinuria (g 24 h$^{-1}$) | 7.1 | 42.4 | 10.2 | 3.4 | 4.1 | 20 | 13.54 | 5.6 | 5.76 | 13.25 | 7.3 |
| Albumin (g l$^{-1}$) | 30 | 20 | 17 | 23 | 26 | 18 | 20 | 28 | 21 | 12 | 29 |

LC, light chain; Ig, immunoglobulin.

proteinuria, serum albumin, glomerular filtration rate and blood counts. The disease burden/organ dysfunction in the MP and VAD groups appears similar (Table 1) with good preservation of bone marrow (median haemoglobin 13.9 vs 13.6 g dl$^{-1}$) and renal function (median serum creatinine 113 vs 100 μmol l$^{-1}$) and degree of nephrotic syndrome (median UTP 7.25 vs 8.6 g and serum albumin 23 vs 21 g l$^{-1}$) respectively. One patient in each of the VAD and MP groups had symptomatic cardiac failure with cardiac amyloid infiltration confirmed at post mortem. The remaining patients had no electrocardiographic, echocardiographic or isotope evidence of impaired cardiac function.

The interval between diagnosis and treatment was 6–65 (median 6) and 40–497 (median 138) days for the VAD- and MP-treated group respectively. The VAD group had a lower performance status and was deemed to require immediate treatment. The response rates and survival characteristics are shown in Table 2. Three out of four patients achieved PR with VAD and are alive and in remission 4.1, 6.5 and 9.3 years (median survival of group 64.5 months) from diagnosis, whereas those treated with supportive care survived for a median of 11.7 months with no spontaneous responses. Two of seven patients treated with MP alone achieved PR, of whom only one is alive and in continuing PR at 3.6 years; the second died of refractory anaemia with excess of blasts in transformation (RAEBt) after 7.1 years. The median survival in this group was 17.7 months. One patient in each treatment group died of gastrointestinal haemorrhage related to autopsy-proven gastric amyloid. When VAD was commenced after failure of MP in two patients no response was achieved, although one patient remains alive at 13 months having received three cycles of VAD, the other died of gastrointestinal haemorrhage after three cycles of VAD.

In this group of patients with amyloid treated with VAD, the toxicity was similar to that seen when patients with myeloma are treated with VAD.

Treatment of patients with poor prognostic features can be successful: a 49-year-old man with a performance status of 40% (Karnofsky et al, 1948) was admitted with a UTP of 42.4 g, a serum albumin of 20 g l$^{-1}$ and a creatinine clearance of 23 ml min$^{-1}$. A bone marrow aspirate revealed 14% plasma cells and AL was confirmed on renal, rectal and bone marrow biopsies. [$^{123}$I]SAP (serum amybid p component) scintigraphy confirmed heavy amyloid deposition in liver, spleen, kidneys and bone marrow (Hawkins et al, 1988). The patient was treated with six cycles of VAD, followed by 6-weekly oral melphalan and prednisolone (MP) and subsequently $\alpha_{2b}$-interferon. Improvement was rapid with a 50% reduction in serum monoclonal protein concentration and urinary total protein after six cycles of VAD and only three of melphalan and prednisolone (322 days). Partial response was achieved after 3.9 years. The patient remains in continuing PR with a good performance status and a daily UTP < 3 g 4 years later and a recent SAP scan showed only minor abnormalities of kidneys, liver and spleen with continuing improvement since commencing interferon. The other two VAD responders had a reduction in UTP from 7.1 and 3.4 to < 0.05 g. The latter also had hepatic involvement, which resolved completely (disappearance of hepatomegaly and normalization of deranged liver function tests).

### DISCUSSION

These preliminary data support the further investigation of VAD as the initial treatment regimen for AL. Treatment was not allocated by randomization and there are clearly differences between the two groups. For example, more patients fulfilled the diagnostic criteria for myeloma (Salmon and Cassady, 1993) in the VAD-treated group. The presence or absence of myeloma is not, however, a prognostic factor for survival in the first year after diagnosis (Kyle and Gertz, 1995). Both sets of patients had similar median age, performance status, proteinuria, hypoalbuminaemia

© Cancer Research Campaign 1998

**Table 2** Response rates and survival characteristics

| | VAD | MP | Best supportive care |
|---|---|---|---|
| Partial response rate | 3 of 4 | 2 of 7 | 0 of 18 |
| Median time to partial response (days) (absence of M-protein and UTP < 3 g) | 357 | 659 | |
| Median survival from diagnosis (days) | 1935 | 530 | 395 |
| Median survival from treatment (days) | 1932 | 392 | |

and renal impairment. Cardiac amyloid is a poor prognostic factor (Gertz et al, 1991; Kyle and Gertz, 1995; Skinner et al, 1996) and there was one patient in each treatment group. The PR rate in three of four VAD-treated patients is interesting and includes the most severely affected patient. The response rate in those receiving MP (two of seven) was similar to the response rates in published series from randomized trials (Kyle and Greipp, 1983; Kyle et al, 1985, 1997; Marione et al, 1994; Merlini et al, 1995). The fact that VAD was followed by MP makes interpretation difficult. The time to response was shorter in those treated with VAD than MP alone (median 357 and 659 days to PR respectively) and all three exhibited evidence of response before commencement of MP. The survival of VAD-treated patients is better than that of melphalan-treated patients and control subjects treated with supportive care.

We believe that this report will be of interest to clinicians who treat AL. We have shown that VAD can be used in patients of poor performance and it may have an advantage in patients with moderate to severe renal impairment, as the drugs are not renally excreted. Aggressive myeloablative therapy with high-dose melphalan has recently been shown to be possible in some patients (Comenzo et al, 1996). Patients with NS caused by AL should be considered for chemotherapy. Prospective randomized trials are required to assess the benefits of newer regimens, such as VAD, over conventional treatment with melphalan and prednisolone.

### ABBREVIATIONS

AL, primary (light-chain) amyloid; MP, melphalan and prednisolone; NS, nephrotic syndrome; PR, partial response; SAP, serum amyloid P component; UTP; 24-h urinary total protein; VAD, vincristine, doxorubicin and dexamethasone combination chemotherapy.

### ACKNOWLEDGEMENTS

We would like to thank Professor MB Pepys and Dr. PN Hawkins at the Hammersmith Hospital for performing [$^{123}$I]SAP scans.

### REFERENCES

Anderson H, Scarffe JH, Ranson M, Young R, Wieringa GS, Morgenstern GR, Fitzsimmons L and Ryder D (1995) VAD chemotherapy as remission induction for multiple myeloma. *Br J Cancer* **71**: 326–330

Case DC, Lee BJ and Clarkson BD (1977) Improved survival times in multiple myeloma treated with melphalan, prednisolone, cyclophosphamide, vincristine and BCNU: M-2 protocol. *Am J Med* **63**: 897–903

Comenzo RL, Vosburgh E, Simms RW, Bergethon P, Sarnacki D, Finn K, Dubrey S, Faller DG, Falk RH and Skinner M (1996) Dose-intensive melphalan with blood stem cell support for the treatment of AL amyloidosis: one-year follow-up in five patients. *Blood* **88**: 2801–2806

Fielder K and Durie BG (1986) Primary amyloidosis associated with multiple myeloma. Predictors of successful therapy. *Am J Med* **80**: 413–418

Gertz MA and Kyle RA (1989) Primary systemic amyloidosis – a diagnostic primer. *Mayo Clin Proc* **64**: 1505–1519

Gertz MA, Kyle RA and Greipp PA (1991) Response rates and survival in primary systemic amyloidosis. *Blood* **77**: 257–262

Hawkins PN, Myers MJ, Lavender JP and Pepys MB (1988) Diagnostic radionuclide imaging of amyloid: biological targeting by circulating serum amyloid P component. *Lancet* **1**: 1413–1418

Karnofsky DA, Abelman WH, Craver LF and Burchenal JH (1948) The use of nitrogen mustards in the palliative treatment of carcinoma. *Cancer* **1**: 634–656

Kyle RA and Gertz MA (1995) Primary systemic amyloidosis: clinical and laboratory features in 474 cases. *Semin Haematol* **32**: 45–59

Kyle RA and Greipp PR (1983) Amyloidosis (AL): clinical and laboratory features in 229 cases. *Mayo Clin Proc* **58**: 665–683

Kyle RA, Greipp PR, Garton JP and Gertz MA (1985) Primary systemic amyloidosis. Comparison of melphalan/prednisolone versus colchicine. *Am J Med* **79**: 708–716

Kyle RA, Gertz MA, Greipp PR, Witzig TE, Lust AL, Lacy MQ and Therneau TM (1997). A trial of three regimens for primary amyloidosis: colchicine alone, melphelan and prednisone and melphelan, prednisone and colchicine. *New Engl J Med* **336**: 1202–1207

Levy YD, Belghiti Deprez D and Sobels A (1988) [Treatment of AL amyloidosis without myeloma]. *Ann Med Interne Paris* **139**: 190–193

Linke RP, Nathrath WBJ and Eulitz M (1986) Classification of amyloid syndromes from tissue sections using antibodies against various amyloid fibril proteins: report of 142 cases. In *Amyloidosis* Glenner GG, Osserman EF, Benditt EP, et al (eds) pp. 599. Plenum: New York

Marione GS, Quaglini S and Bellotti V (1994) AL amyloidosis: clinical and therapeutic aspects of an Italian study protocol. In: *Amyloid and Amyloidosis 1993*, Kisilevsky R, Benson MD and Frangione B (eds) pp. 206–208. Parthenon: Park Ridge, NJ.

Merlini G, Ascari E, Amboldi N, Bellotti V, Arbustini E, Perfetti V, Ferrari M, Zorzoli I, Marinone MG, Garini P, Diegoli M, Trizio D and Ballinari D (1995). Interaction of the anthracycline 4′-iodo-4′-deoxydoxorubicin with amyloid fibrils: inhibition of amyloidogenesis. *Proc Natl Acad Sci USA* **92**: 2959–2963.

Perfetti V, Bellotti V, Maggi A, Garini P and Merlini G (1994). Amyloidogenic plasma cell precursors identified by anti-idiotypic monoclonal antibodies: phenotypical and functional characterisation. In: *Amyloid and Amyloidosis 1993*, Kisilevsky R, Benson MD and Frangione B (eds) pp. 299–301. Parthenon: Park Ridge, NJ.

Salmon SE and Cassady JR (1993) Plasma cell neoplasms. In: *Cancer: Principles and Practice of Oncology*, DeVita VT, Hellman S and Rosenberg SA (eds) pp. 1984–1985. Lippincott: Philadelphia, PA

Skinner M, Anderson J, Simms R, Falk R, Wang C, Libbey C, Jones LA and Cohen AS (1996) Treatment of 100 patients with primary amyloidosis: a randomized trial of melphalan, prednisolone and colchicine versus colchicine only. *Am J Med* **100**: 290–298

© Cancer Research Campaign 1998